# Inhibition of HIV-1 infection by aqueous extracts of *Prunella vulgaris *L.

**DOI:** 10.1186/1743-422X-8-188

**Published:** 2011-04-23

**Authors:** ChoonSeok Oh, Jason Price, Melinda A Brindley, Mark P Widrlechner, Luping Qu, Joe-Ann McCoy, Patricia Murphy, Cathy Hauck, Wendy Maury

**Affiliations:** 1 Department of Microbiology, University of Iowa, Iowa City, IA 52242, USA; 2 U.S. Department of Agriculture-Agricultural Research Service, North Central Regional Plant Introduction Station, Ames, IA 50011, USA; 3 Department of Food Science and Human Nutrition, Iowa State University, Ames, IA 50011, USA; 4 Department of Pediatrics, Emory University School of Medicine and Children's Healthcare of Atlanta, Atlanta, Georgia 303221, USA; 5 Bent Creek Institute/NCSU, The North Carolina Arboretum, 100 Frederick Law Olmsted Way, Asheville, NC 28806, USA

**Keywords:** human immunodeficiency virus, HIV, antiviral, microbicide, plant extract, self-heal

## Abstract

**Background:**

The mint family (Lamiaceae) produces a wide variety of constituents with medicinal properties. Several family members have been reported to have antiviral activity, including lemon balm (*Melissa officinalis *L.), sage (*Salvia *spp.), peppermint (*Mentha *× *piperita *L.), hyssop (*Hyssopus officinalis *L.), basil (*Ocimum *spp.) and self-heal (*Prunell*a *vulgaris *L.). To further characterize the anti-lentiviral activities of *Prunella vulgaris*, water and ethanol extracts were tested for their ability to inhibit HIV-1 infection.

**Results:**

Aqueous extracts contained more anti-viral activity than did ethanol extracts, displaying potent antiviral activity against HIV-1 at sub μg/mL concentrations with little to no cellular cytotoxicity at concentrations more than 100-fold higher. Time-of-addition studies demonstrated that aqueous extracts were effective when added during the first five hours following initiation of infection, suggesting that the botanical constituents were targeting entry events. Further analysis revealed that extracts inhibited both virus/cell interactions and post-binding events. While only 40% inhibition was maximally achieved in our virus/cell interaction studies, extract effectively blocked post-binding events at concentrations similar to those that blocked infection, suggesting that it was targeting of these latter steps that was most important for mediating inhibition of virus infectivity.

**Conclusions:**

We demonstrate that aqueous *P. vulgaris *extracts inhibited HIV-1 infectivity. Our studies suggest that inhibition occurs primarily by interference of early, post-virion binding events. The ability of aqueous extracts to inhibit early events within the HIV life cycle suggests that these extracts, or purified constituents responsible for the antiviral activity, are promising microbicides and/or antivirals against HIV-1.

## Background

*Prunella vulgaris*, commonly known as "self-heal" or "heal-all", is a low-growing perennial herb with worldwide distribution. It is a member of the mint family Lamiaceae that has been used to treat wounds, inflammation, and other minor body disorders across multiple traditional cultures [1-4].

Bioactive compounds are plentiful in both aqueous and ethanol *P. vulgaris *extracts. Aqueous extracts contain abundant polyphenols and complex carbohydrates, whereas more hydrophobic metabolites, such as triterpenes and flavonoids along with some polysaccharides and polyphenols, are found in ethanol extracts [5-7]. *Prunella vulgaris *polysaccharides have pro-inflammatory activities in a macrophage cell line [8] and immunomodulatory activity in other tumor lines [9], whereas several of its triterpenes contain significant anti-inflammatory activity [10]. Large quantities of antioxidants are known to be present in aqueous *P. vulgaris *extracts with the polyphenolic compound, rosmarinic acid, being one of the most abundant [6,11].

*P. vulgaris *extracts are reported to have antiviral and anti-bacterial properties, although constituents responsible for these activities are incompletely characterized to date [6,12,13]. Anionic polysaccharides in aqueous extracts of *P. vulgaris *can decrease the replication of herpes simplex virus-1 and -2 (HSV-1, HSV-2) by preventing virus binding to cells [12,14-16].

Extracts have also been shown to contain activity against the lentiviruses, HIV-1 and equine infectious anemia virus (EIAV). Our earlier studies investigating the ability of aqueous *P. vulgaris *extracts to inhibit EIAV indicated that both virus binding and early post-binding events were inhibited [17]. Previous studies have identified *P. vulgaris *extract inhibition of a number of steps within the HIV life cycle including: virus binding [18], fusion [19], reverse transcription [13], integration [20], and protease activity [21]. While these steps of the life cycle may be inhibited in vitro, identification of which step(s) are inhibited during HIV infection remain poorly elucidated. For instance, rosmarinic acid extracted from other botanicals has proven effective against HIV-1 integrase in an in vitro assay [5]; however, this polyphenol is not responsible for the anti-retroviral activities of *P. vulgaris *extracts [22-24]. Additional members of the Lamiaceae, such as peppermint (*Mentha *× *piperita *L.) and lemon balm (*Melissa officinalis *L.), are also known to have anti-viral activities, but specific constituents responsible for those activities remain unidentified [7,14].

To date, isolation and identification of *P. vulgaris *compounds inhibiting HIV-1 is limited to a sulfated polysaccharide called Prunellin [5]. This constituent is likely the same water-soluble, 10 kDa anionic constituent that was demonstrated to interfere with HIV-1 virion binding to permissive cells by blocking CD4 interactions [18].

In our studies, evaluation of water and ethanol extracts from several *P. vulgaris *accessions demonstrated better anti-HIV activity in aqueous extracts than in ethanol extracts, with 50% inhibition of HIV infectivity (IC_50_) at about 0.8 μg/mL of aqueous extracts. Aqueous extracts from *P. vulgaris *Ames 27664 and 27748 were found to be about 35 times more potent against HIV-1 infectivity than against EIAV [17]. This extract inhibited early events in the HIV-1 life cycle. As others have reported [18], our findings also indicated that aqueous extracts blocked HIV-1 binding to permissive cells; however, this blockage was incomplete with a maximum of ~40% inhibition at extract concentrations that were more than an order of magnitude higher than the IC_50_, suggesting that binding interference was not primarily responsible for the observed antiviral activity. Instead, we found that post-binding entry events were strongly inhibited with an IC_50 _of about 1 μg/mL, a value similar to that found when extract was added at the initiation of infection. Thus, we propose that inhibition of post-binding entry events accounts for the preponderance of the antiviral activity of *P. vulgaris *against HIV-1.

## Materials and methods

### Growth and collection of *P. vulgaris *accessions

All *P. vulgaris *plant samples were provided by the North Central Regional Plant Introduction Station (NCRPIS, Ames, IA) of the U.S. Department of Agriculture - Agricultural Research Service. Accessions utilized in experiments were produced in Ames, IA and grown from seeds of populations collected in the U.S.A. and South Ossetia, Republic of Georgia. Accession details are provided in Table 1. Both seed and voucher specimens were collected from natural populations, and specimens were keyed to species [25,26]. Seeds from accessions were germinated in Petri plates at 25°C, transferred to flats in a greenhouse (20-25°C) before final field transfer into individual control pollinated screened cages in Ames, IA. Upper flowering portions of 14-month-old plants were harvested, dried for 1 week at 38°C in a forced-air dryer with constant humidity and ground for analysis. Dried samples were stored in sealed plastic bags overlayed with nitrogen gas at -20°C until use. All voucher specimens representing both original and regenerated populations are stored in the Ada Hayden Herbarium, Iowa State University (Ames, IA: ISC). Seeds representing both original and regenerated populations are stored at the NCRPIS under controlled conditions (-20°C; 4°C for regenerated samples). Information about the specific provenance of all accessions used for the experiments is available via the Germplasm Resources Information Network database at http://www.ars-grin.gov/npgs/acc/acc_queries.html.

**Table 1:** Provenance of *P. vulgaris *accessions used in this study

Accession	Geographic Origin	Habitat
Ames 27664	North Carolina, USA	Lakeside along pine-oak forest

Ames 27665	North Carolina, USA	Roadside in spruce-fir forest

Ames 27666	North Carolina, USA	Trailside, rich mesic cove forest

Ames 27748	Missouri, USA	Roadside along prairie remnant, partly mowed

Ames 28312	Iowa, USA	Muddy, rocky bed of Rock Creek

Ames 28355	Iowa, USA	Des Moines River floodplain

Ames 28356	Iowa, USA	Slump below sandstone cliff

Ames 28358	Iowa, USA	Cleared woods

Ames 28359	Iowa, USA	Springs in dense forest

Ames 28313	Iowa, USA	Mesic prairie

Ames 29161	S. Ossetia, Republic of Georgia	Pine-spruce forest edge

Ames 29156	S. Ossetia, Republic of Georgia	Roadside along Tana River

Ames 29160	S. Ossetia, Republic of Georgia	Alpine meadow

Ames 29155	S. Ossetia, Republic of Georgia	Roadside along secondary subalpine meadow

PI 656839	Iowa, USA	Native prairie remnant

PI 656840	Iowa, USA	Shoreline of pond

PI 656841	Iowa, USA	Bottom ground field

PI 656842	Missouri, USA	Roadside prairie remnant

### Extraction and fractionation of *P. vulgaris*

All glassware was heated at 200°C for 2 hours to destroy endotoxin [27].

#### Water extraction

Boiling, endotoxin-free water was poured over dried *P. vulgaris *at a ratio of 100 mL/6 g of dried tissue. The plant material was steeped, with constant stirring, for 1 hour and filtered through a G6 glass fiber circle (Fisher Scientific) in a Buchner funnel. The filtrate was centrifuged at 10,000 × g for 20 minutes to remove any additional particulates. The extract was lyophilized, weighed, and re-dissolved in either DMSO or sterile endotoxin-free water.

#### Ethanol extraction

Six g of dried *P. vulgaris *was extracted with 500 mL of 95% ethanol via Soxhlet for 6 hours. The extract was filtered, dried by rotary evaporation at <40°C and then lyophilized. Extracts were resuspended in DMSO.

### Endotoxin levels of extracts and fractions

All extracts and fractions were evaluated for endotoxin by using the Chromogenic Limulus Amebocyte Lysate Test kit per manufacturer's instructions (Cambrex Bioscience Inc.). This assay is able to detect concentrations of endotoxin of ≥0.07 EU/mL. All extracts had <0.07 EU/mL at the highest concentrations used in these studies.

### Cells and viral strains

HeLa37 cells were maintained in high glucose DMEM with 10% fetal calf serum (FCS). These cells ectopically express CD4 and CCR5 and endogenously express CXCR4 [28]. All media were supplemented with penicillin and streptomycin.

Stocks of HIV-1 were generated by transient transfections of 293T cells. The molecular clones, pNL4-3 [29], pAd8 [30] or p256 [31], were transfected into 80% confluent 15 cm plates of 293T cells using either a calcium phosphate protocol or a PEI lipofection protocol as described [32]. Supernatants were collected at 48 hours following transfection, filtered through a 0.45 μ filter, distributed into 500 μL aliquots and stored at -80°C. Viral titers were determined by infection of HeLa37 cells with the single round of infection assay as previously described [33].

### Viral infection and time-of-addition studies

#### Inhibition of infectivity studies

Approximately 200 infectious HIV-1 particles were combined with the concentrations of extracts as noted. The amount of vehicle was adjusted so that equivalent concentrations of vehicle were present in all wells within an experiment. In studies that used extracts resuspended in DMSO, DMSO concentrations never exceeded 0.5%. The extract and virus mixture was added to 2 × 10^4 ^cells/well of HeLa37 cells in a 48-well format, resulting in a multiplicity of infection (MOI) of ~0.01. The cells were maintained for 40 hours. Cells were fixed with 75% acetone/25% water and immunostained for HIV antigens, as previously described [33], with human anti-HIV antisera. HIV antigen-positive cells within the infected cell monolayer were counted and titers determined. IC_50 _and IC_90 _concentrations were determined with TableCurve software (Systat Academic).

To quantify the number of logs of virus infectivity inhibited by the extract, approximately 1.15 × 10^4 ^infectious particles of NL4-3 were incubated at room temperature for 10 minutes with various concentrations of *P. vulgaris *accession Ames 27748 aqueous extract. Following the incubation, serial dilutions of the incubated virus were added to 2 × 10^4 ^HeLa37 cells in a 48-well format and appropriate concentrations of extract on the cells were maintained for the duration of the experiment. Cells were fixed at 40 hours following infection and immunostained as described above. Wells with serial dilutions containing between 10 and 250 virus positive cells were counted and back-calculations made to obtain the numbers of infectious units of virus/mL.

#### Virion-stability studies

Sucrose step gradients were prepared by layering 250 μL aliquots of decreasing concentrations of sucrose (20%-60%) into 3 mL ultra centrifugation tubes. The gradients were allowed to equilibrate at 4°C for 3 hours. Virions were treated with 132 μg/mL of aqueous Ames 27664 extract or 126 μg/mL of aqueous Ames 27748 extract, 0.5% Triton-X 100 or 0.4% DMSO for 1 hour at 37°C and loaded onto the top of the gradients. Tubes were centrifuged for 16 hours at 40,000 rpm in a SW60 rotor at 4°C and stopped without a brake. Two hundred and fifty μL aliquots were collected beginning from the top of the tube and stored at -80°C until analyzed by immunoblotting.

#### Time of addition studies

Approximately 200 infectious particles of NL4-3 were added to wells containing 2 × 10^4 ^HeLa37 cells (MOI = ~0.01). Extracts of *P. vulgaris *Ames 27748 (10 μg/mL) or vehicle were added to triplicate wells every hour beginning at 2 hours prior to infection and continuing until 8 hours after initiation of infection. Forty hours following infection, the cells were fixed, immunostained for HIV antigens, and the HIV-1 positive cells counted.

#### Extract/cell pre-incubation studies

Extract (0.1 to 50 μg/mL) was incubated in growth media at 37°C with 2 × 10^4 ^cells/well of HeLa37 cells in a 48-well format for 1 hour. At the end of the incubation, media containing the extract was removed, the cells were washed in 1× PBS twice, and refreshed with growth media. Approximately 250 infectious HIV-1 particles were added to the treated cells resulting in a MOI of 0.012. Cells were fixed at 40 hours following infection and immunostained as described above.

#### Extract/virion pre-incubation studies

Approximately 4 × 10^4 ^infectious HIV-1 particles were combined with extracts (0.3 to 30 μg/mL) and incubated at room temperature for 10 minutes. The mixture was diluted 100 fold with media to reduce extract concentrations to irrelevant levels and added to 2 × 10^4 ^cells/well of HeLa37 cells in a 48-well format, resulting in a final MOI of 0.02. Cells were fixed at 40 hours following infection and immunostained as described above.

#### Binding studies

HIV-1 NL4-3 (3.8 × 10^5 ^infectious particles) was incubated with 2 × 10^4 ^HeLa37 cells at 4°C for 1 hour (MOI = 19) in the presence of growth media (DMEM with 10% FCS) and increasing extract concentrations. This incubation step permitted virus/cell binding, but prevented virion internalization. Unbound virus was removed by washing the cells several times with growth media, and washed cells were then lysed with 1% SDS. The cell lysates were separated on a 4-20% SDS PAGE and proteins transferred to PVDF membrane. Membranes were probed for HIV capsid (rabbit antibody to p24; #4250 NIH AIDS & Reference Reagent Program) and HRP-conjugated mouse anti-human β-actin monoclonal antibody (Abcam, Cambridge, MA).

#### Post-binding inhibition studies

HIV-1 NL4-3 (2 × 10^5 ^particles) was bound to 2 × 10^4 ^HeLa37 cells (MOI = 10) at 4°C for 2 hour in DMEM with 10% FCS to permit binding, but prevent virion internalization. Media and unbound virus were removed, and cells were refreshed with growth media containing increasing extract concentrations and shifted to 37°C. Forty hours following infection, the cells were fixed, immunostained for HIV antigens, and HIV-positive cells counted.

### Cell-viability studies

Cells were plated and treated with extracts as described above. Forty hours following treatment, cell viability was monitored by ATPLite Assay (Packard Biosciences) per manufacturer's instructions.

### Statistical analysis

Studies were performed at least three independent times, except where noted in the figure legends. Means and standard errors of the mean are shown. Student's t-test was used to evaluate the statistical differences between control and experimental treatments, utilizing two-tailed distribution. A significant difference was determined by a p-value of < 0.05 and significance is identified in each figure. If the p-value was >0.05, the data were not considered statistically significantly different.

## Results

### *Prunella vulgaris *aqueous extracts inhibit HIV infectivity without significant cell toxicity

**Figure 1:**
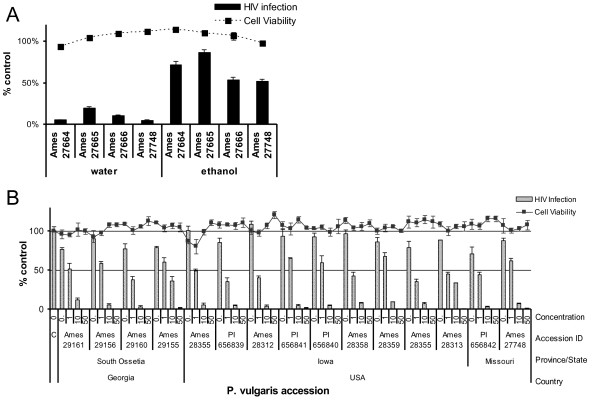
**Aqueous extracts of *P. vulgaris *inhibit HIV-1 infectivity**. **A) **DMSO, water extracts and ethanol extracts of *P. vulgaris *were diluted in media to 0.2% (water extracts: 66 μg/mL of Ames 27664, 42.2 μg/mL of Ames 27665, 59.6 μg/mL of Ames 27666, or 62.4 μg/mL of Ames 27748 and ethanol extracts: 66.8 μg/mL of Ames 27664, 69.2 μg/mL of Ames 27665, 64.2 μg/mL of Ames 27666, or 67.4 μg/mL of Ames 27748). Equivalent numbers of HIV-1 NL4-3 virions were added to each well of HeLa37 cells along with the diluted extracts. Forty hours following infection, cells were fixed and immunostained for viral antigens. Cell-viability studies were performed in parallel in uninfected HeLa37 cells and are shown by the hatched line. Cell viability and virus infectivity are shown as a ratio of the values in the presence of the extracts divided by the DMSO control. Shown are the averages and standard errors of three experiments performed in triplicate. **B) **The anti-HIV-1 dose response curve of 14 aqueous extracts of *P. vulgaris *accessions collected from the U.S. and the Republic of Georgia. All plant material was grown at NCRPIS, Ames, IA. Concentrations of the aqueous extracts noted were mixed with equivalent numbers of NL4-3 virions and added to HeLa37 cells as described in A. Cytotoxicity of the extracts are shown in parallel as the black line. Data are represented as the means and standard errors of the mean of two experiments performed in triplicate.

Water and ethanol extracts were prepared from four accessions of *P. vulgaris*. These extracts were screened for their ability to inhibit virus generated from the well-described, X4-tropic lab molecular clone of HIV-1, pNL4-3, in a single-round infection assay in HeLa37 cells. Extracts and virus were diluted in media and immediately incubated with cells. Forty hours following infection, the cells were fixed, immunostained for expression of HIV antigens, and antigen-positive cells enumerated to determine the level of viral infection (Figure 1A). Although both ethanol and water extracts demonstrated some inhibition, all four water extracts contained significantly more anti-viral activity than did their respective ethanol counterparts. At the concentrations tested, all extracts had little or no cytotoxicity.

Aqueous extracts from 14 additional accessions of *P. vulgaris *from the northern hemisphere were also assessed for anti-HIV-1 activity. All extracts abrogated virus infection at 50 μg/mL with little cytotoxicity (Figure 1B). At 1 to 10 μg/mL, some extracts appeared to be somewhat more inhibitory than others, with HIV-1 replication inhibited by 33 to 66% at 1 μg/mL of the different extracts. As aqueous extracts of Ames 27664 and 27748 displayed some of the strongest anti-viral effects, these extracts were used for further study.

**Figure 2:**
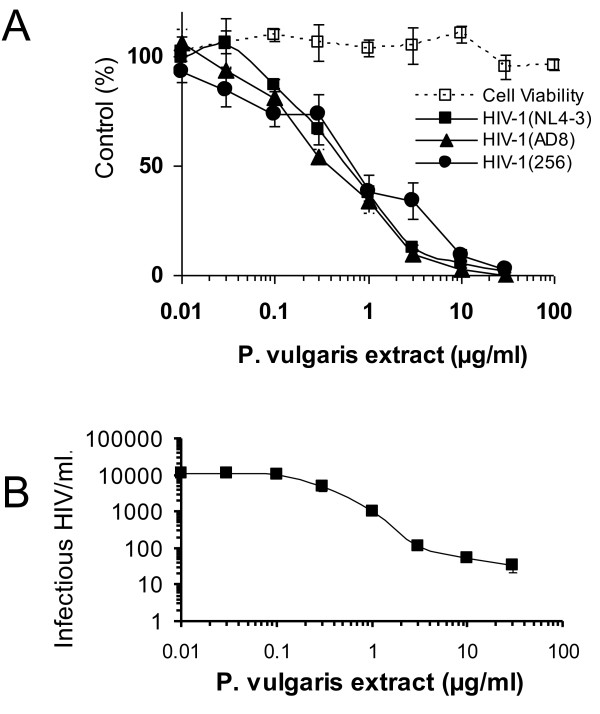
**Aqueous *P. vulgaris *extracts inhibit both X4 and R5 HIV-1**. **A) **Dose response curves of extract inhibition of X4 virus NL4-3 (squares), R5 virus AD8 (triangles) and dual tropic 256 virus (circles). Cytotoxicity of extract was performed in parallel in uninfected cells (open squares). Infections were maintained for forty hours. Cells were fixed and immunostained for HIV antigens and number of HIV antigen-positive cells enumerated. Data are presented as the percent of control infections that did not have extract added. Shown are the averages and standard errors of three experiments performed in triplicate. **B) **Log-log plot of HIV-1 NL4-3 replication in the presence of aqueous *P. vulgaris *extract. Extract was incubated with 1.15 × 10^5 ^infectious virions and the mixture serially diluted in media containing appropriate amounts of extract and added to HeLa37 cells. Infected cells were maintained for 40 hours, fixed and immunostained for HIV antigens as described in A. Shown are the averages and standard errors of three experiments performed in triplicate.

These aqueous extracts were evaluated for their inhibition of not only X4-tropic HIV-1, such a NL4-3, but also for the R5-tropic strain, AD8, and the dual-tropic strain, 256 [29-31]. We found that the extracts effectively inhibited all three HIV-1 strains with similar concentrations inhibiting 50% of virus infectivity (IC_50 _= ~0.8 μg/mL) (Figure 2A). Those concentrations that inhibited 90% of infectivity (IC_90_) varied somewhat from about 3 μg/mL for NL4-3 and AD8 to 11 μg/mL for 256. No extract cytotoxicity was observed at concentrations as high as 100 μg/mL, concentrations that completely abrogated virus infectivity. Accurate cytotoxicity curves and the generation of cytotoxicity concentration_50 _and _90 _values (CC_50 _and CC_90_) could not be obtained in these studies, since significant killing of the monolayer was not achieved. Consequently, we were unable to determine the selectivity index (CC_50_/IC_50_) of *P. vulgaris *Ames 27748 aqueous extracts; however, our studies indicate a robust window between the two values.

To determine the logs of reduction in particle infectivity by *P. vulgaris *Ames 27748 extracts, we incubated 1.15 × 10^5 ^infectious virions of NL4-3 with 0.03 to 30 μg/mL of aqueous extract. Virions were serially diluted in media maintaining appropriate concentrations of extract and added to HeLa37 cells. Virus infectivity was evaluated 40 hours later. Incubation of virions with aqueous extract reduced virion infectivity ~140-fold, an inhibition similar to that reported for a number of clinically relevant antivirals (Figure 2B) [34].

### *Prunella vulgaris *extracts inhibit early steps in the HIV-1 life cycle

**Figure 3:**
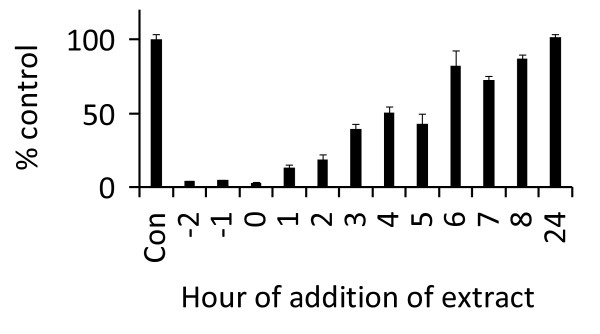
**Time-of-addition studies**. HIV-1 NL4-3 virions (MOI = 0.01) were added to HeLa37 cells at time zero. A final concentration of 10 μg/mL of *P. vulgaris *Ames 27748 extract was added to the cultures at times noted and extract was maintained on the cells for the remainder of the infection. Cells were fixed and immunostained for viral antigens at 40 hours following initiation of the experiment. Data are represented as the percent of control wells (Con) that did not have extract added. Each experiment was performed in triplicate and independently performed three times. Shown are the mean and standard error of the mean for each time point.

To identify the step(s) during the viral life cycle that are inhibited by the aqueous extracts, time-of-addition experiments were performed. HeLa37 cells were infected with NL4-3 at time zero. At times noted in Figure 3, a final concentration of 10 μg/mL of aqueous *P. vulgaris *extract was added to each well, and the infection continued for a total of 40 hours. Addition of extracts at the initiation of infection resulted in >95% inhibition of HIV-1 infection. Adding the extract one hour following initiation of infection reduced the inhibitory activity of the extract slightly to 87% inhibition and by 4 hours the extract was <50% as effective as when it was added at the initiation of infection (Figure 3). Studies have shown that reverse transcription of the HIV-1 genome is initiated at ~5 hours following infection and requires several further hours for complete genomic DNA synthesis [35,36]. Thus, the extract must be inhibiting one or more steps of the HIV-1 life cycle prior to reverse transcription.

**Figure 4:**
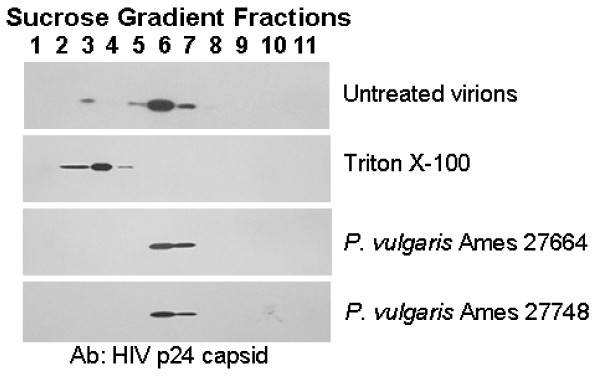
**Density of HIV-1 is not altered by aqueous *P. vulgaris *extract**. HIV NL4-3 virions were untreated or treated with 0.5% Triton X-100, 132 μg/mL of aqueous extracts of Ames 27664 or 126 μg/mL of Ames 27748 for 1 hour at 37°C. Virus was applied to the top of a 20-60% sucrose gradient and ultracentrifuged. Fractions were collected, separated on SDS PAGE and transferred to nitrocellulose. The blot was probed with an antibody against HIV-1 p24.

To further dissect the mechanism of aqueous *P. vulgaris *extract inhibition, we assessed whether the extracts were altering the integrity of viral particles, thereby rendering them non-infectious. Previous studies with extracts from other Lamiaceae species demonstrated that botanical constituents alter the density of virions, suggesting that components of the extract were binding to the virus particles and perhaps inactivating them [7]. HIV-1 NL4-3 was incubated with a final concentration of 132 μg/mL of aqueous extract of Ames 27664 or 126 μg/mL of Ames 27748 extract for one hour at 37°C. As a control, 0.5% Triton X-100 was added to virions to lyse the viral particles. Samples were separated on a discontinuous sucrose gradient by ultracentrifugation. Fractions were collected and analyzed for HIV-1 capsid p24 by immunoblotting, thereby assessing the location of a virion-associated HIV protein within the sucrose gradient. Capsid protein was present primarily in fractions 5-7 of untreated samples, whereas Triton X-100 shifted capsid nearer the top of the gradient, indicating lysis of the virions (Figure 4). The location of capsid remained in fractions 6 and 7 in the presence of high concentrations of either extract, demonstrating that virions remain intact in the presence of the extract. This finding also suggested that the extracts were not heavily coating the virions, thereby altering their density.

**Figure 5:**
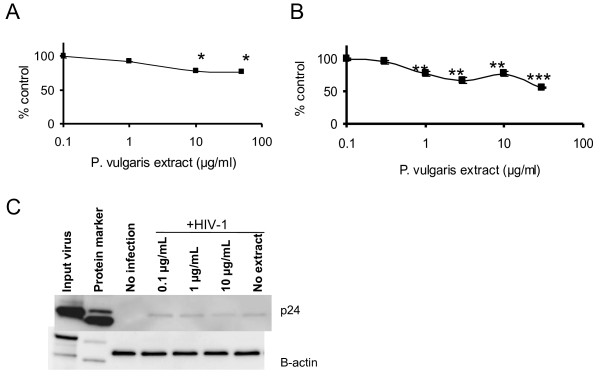
***P. vulgaris *extracts have a modest effect on HIV-1 binding to permissive cells**. **A) **Pre-incubation of extract with cells. HeLa37 cells were pre-incubated with concentrations of aqueous extracts noted in the panel in DMEM with 10% FCS for 1 hour at 37°C. Unbound extracts were removed and cells were suspended in fresh media and 200 infectious units of HIV-1 NL4-3 added to the culture (MOI = 0.01). Cells were fixed and immunostained for HIV antigens 40 hours after infection. Pre-exposure of cells to extract reduced the level of virus infectivity by 20%, which was statistically significantly different from the control. *, p < 0.05. **B) **Pre-incubation of extracts with virions. Approximately 4 × 10^4 ^infectious HIV-1 NL4-3 particles were combined with extracts and incubated at room temperature for 10 minutes. The mixture was diluted 100 fold with media to reduce extract concentrations to irrelevant levels and added to 2 × 10^4 ^cells/well of HeLa37 cells in a 48-well format, resulting in a final MOI of 0.02. Cells were fixed at 40 hours following infection and immunostained as described above. **, p < 0.01; ***, p < 0.001. **C) **Ability of extracts to inhibit HIV-1 binding to cells. Increasing concentrations of *P. vulgaris *extract were incubated with HIV-1 NL4-3 (3.8 × 10^5 ^infectious particles) (MOI = 19) and 2 × 10^4 ^HeLa37 cells at 4°C for 2 hours in DMEM with 10% FCS. Unbound virus was removed with several washes of media and cells were then lysed. Cell lysates were immunoblotted for HIV-1 p24 and cellular β-actin. All experiments were performed in triplicate and independently performed three times.

We performed three different assays to determine if *P. vulgaris *aqueous extracts altered virion/cell interactions. In our first set of studies, extracts were pre-incubated for 1 hour at 37°C with HeLa37 cells. Unbound extract was removed, media was refreshed, virus was added to the treated cells, and infections were assessed at 40 hours following initiation. Extract/cell pre-incubation had a small, but statistically significant inhibition of HIV infectivity, decreasing infection by about 20% at 10 to 100 μg/mL of extract (Figure 5A). We also assessed if the pre-incubation of extracts with virions affected virus infectivity. When virus was pre-incubated with extract and the mixture was diluted with media 100 fold to achieve irrelevant concentrations of extract prior to addition to cells, partial inhibition of infectivity was achieved with as little as 1 μg/mL and as high as 30 μg/mL, providing evidence that extract and virions interact and this interaction provides partial inhibition of infectivity (Figure 5B). We also directly evaluated if the extract interfered with HIV binding to permissive cells. HIV-1 NL4-3 (MOI = 2) was incubated with HeLa37 cells at 4°C for 2 hours in the presence of increasing concentrations of aqueous extract from *P. vulgaris *Ames 27748. Unbound viruses were removed with several washes, and cells were lysed. Cell lysates were separated by SDS PAGE and transferred to nitrocellulose. Blots were probed for both capsid p24 and β-actin. The presence of 10 μg/mL of extract modestly decreased virus binding to cells, consistent with the decrease in infectivity shown in Figure 5A and 5B (Figure 5C). In total these findings support the possibility that extract blocks HIV binding to cells as others have reported [5,18]. However, inhibition was incomplete even at extract concentrations that were significantly higher than those required to inhibit virus infectivity, suggesting that other steps within the virus life cycle are also inhibited by compounds in the extract.

**Figure 6:**
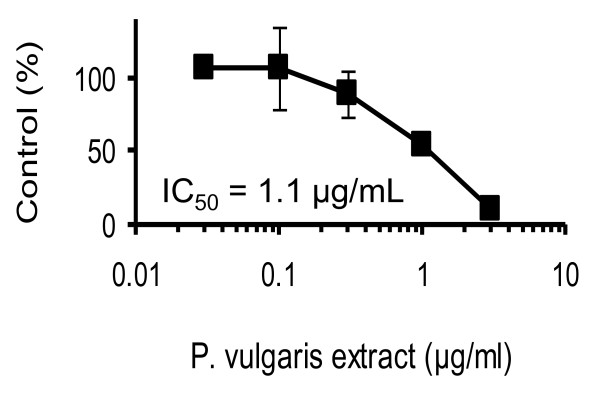
**Aqueous *P. vulgaris *extracts inhibit early post-binding events in the HIV-1 life cycle**. HIV-1 NL4-3 was bound to HeLa37 cells for 2 hours at 4°C. Unbound virus was removed and the cells were refreshed with warmed media containing noted amounts of aqueous extract. Infections were maintained for an additional 40 hours, fixed and immunostained for HIV antigens. Data are represented as the percent of control wells that did not have extract added. Shown are the mean and standard error of the mean for each time point and studies were performed in triplicate three independent times.

To determine if entry events that are downstream from virus binding were inhibited by the extract, NL4-3 was incubated with HeLa37 cells at 4°C for 2 hours to allow virus binding, but prevent internalization. Unbound virus was washed away, prewarmed media containing increasing concentrations of extract were added to the cells, and the cells were then shifted to 37°C for the remainder of the experiment. In these experiments, the extract effectively inhibited HIV infectivity with a similar dose response curve and at a similar IC_50 _concentration to that found when extract was added at the initiation of infection (Figure 6). These findings supported the possibility that post-binding events are inhibited by the extract and that this inhibition is likely to be primarily responsible for the inhibition of HIV-1 infectivity by the *P. vulgaris *extract.

## Discussion

This study explored the antiviral activity of *P. vulgaris *extracts against HIV-1. Aqueous extracts from several accessions demonstrated more robust antiviral activity than did their ethanol counterparts, indicating that polar constituents are important for the antiviral activity. These findings are consistent with previous antiviral observations made with *P. vulgaris *extracts in studies against EIAV and HSV [12,14,16,17]. Time-of-addition studies demonstrated that early events within the life cycle of HIV-1 were inhibited. The extracts reduced virus infectivity by more than two logs and inhibited both R5- and X4-tropic viruses, suggesting that inhibition was not occurring through interference with specific virion interactions with one of the host's chemokine receptors. The extracts were found to inhibit viral entry through at least two different mechanisms. Extracts interfered with HIV-1 virion binding to permissive cells when pre-incubated with either virions or target cells; however, this inhibition was incomplete, maximally producing about 40% inhibition. Consistent with the relatively modest effect that the extract had in these experiments, HIV-1 binding to cells was only partially inhibited by high concentrations (10 μg/mL or more) of extract. In contrast, the addition of extract following pre-binding of HIV-1 to cells was almost as effective at blocking virus infection as the addition of the extract at the time of infection. While we cannot exclude the possibility that the addition of extract is removing pre-bound viruses from the surface cells, our overall findings suggest that the extract principally inhibits HIV-1 infectivity by blocking one or more post-binding entry steps. An earlier study reported that aqueous *P. vulgaris *extracts interferes with six-helix bundle formation [19]. Our findings are consistent with these observations, suggesting that inhibition of virus fusion events may principally be responsible for the inhibition of HIV infectivity by *P. vulgaris*.

Interestingly, our studies with aqueous *P. vulgaris *extracts indicate that this extract has significantly stronger inhibitory activity against HIV-1 than against the related lentivirus EIAV, with HIV-1 inhibition observed with sub-μg/mL concentrations of extracts. Using extracts from the same *P. vulgaris *accessions (Ames 27664 and 27748), EIAV inhibition occurred with an IC_50 _of ~28 μg/mL [17]. As with HIV-1, aqueous extracts inhibited EIAV infection by blocking virus entry, impacting both binding and post-adherence events. Extract fractionation and identification of the bioactive constituents will be required to determine if a single compound, a related group of compounds or multiple, disparate ones are responsible for the anti-lentiviral effects and whether it is the same or different constituents responsible for inhibiting EIAV and HIV-1.

Identification and purification of the bioactive compounds that are present in the aqueous extracts of *P. vulgaris *is ongoing. Preliminary fractionation studies indicated that constituents with quite different solubility properties in ethanol have significant anti-HIV-1 activity, suggesting that multiple antiviral compounds are present in the extract (data not shown). Our studies suggest that both large carbohydrates and tannins may contribute to the anti-HIV-1 activity. To date, a single anti-HIV constituent from *P. vulgaris *has been identified. This is a10 kDa sulfated carbohydrate called Prunellin that inhibits HIV entry [5]. Interestingly, a carbohydrate of approximately that same size was responsible for inhibiting HSV-1 entry into cells [16]. Prunellin may be responsible for the *P. vulgaris *inhibition of binding of these enveloped viruses to cells. Whether it is Prunellin or if other unidentified *P. vulgaris *constituents are responsible for the inhibition of post-binding entry events remains to be determined.

## List of abbreviations

HIV: human immunodeficiency virus; EIAV: equine infectious anemia virus; HSV-1: herpes simplex virus-1; R5: CCR5 utilizing HIV; X4: CXCR4 utilizing HIV; DMSO: dimethyl sulfoxide

## Competing interests

The authors declare that they have no competing interests.

## Authors' contributions

CSO and JP were responsible for all of studies performed and generated preliminary data figures. MB was responsible for oversight and direction of some studies. MPW, LQ and J-AM were responsible for obtaining and characterizing the *Prunella vulgaris *accessions. Furthermore, these individuals planted, maintained, harvested and processed the plant material. CH was responsible for the production of extracts and PM was responsible for the oversight of the production of all extracts. WM was responsible for oversight of the project including design and coordination of the study. In addition, she organized and wrote the manuscript and generated the final figures. All authors have read and approved of the final manuscript.
